# Additive Manufacturing of Binary and Ternary Oxide Systems Using Two-Photon Polymerization and Low-Temperature Sintering

**DOI:** 10.3390/nano14231977

**Published:** 2024-12-09

**Authors:** Halima El Aadad, Hicham El Hamzaoui, Yves Quiquempois, Marc Douay

**Affiliations:** Univ. Lille, CNRS, UMR 8523—Physique des Lasers Atomes et Molécules (PhLAM), F-59000 Lille, France; yves.quiquempois@univ-lille.fr (Y.Q.); marc.douay@univ-lille.fr (M.D.)

**Keywords:** additive manufacturing, two-photon polymerization, multicomponent systems, sol–gel process, low-temperature sintering

## Abstract

Multicomponent oxide systems have many applications in different fields such as optics and medicine. In this work, we developed new hybrid photoresists based on a combination of an organic acrylate resin and an inorganic sol, suitable for 3D printing via two-photon polymerization (2PP). The inorganic sol contained precursors of a binary SiO_2_-CaO or a ternary SiO_2_-CaO-P_2_O_5_ system. Complex microstructures were 3D printed using these hybrid photoresists and 2PP. The obtained materials were characterized using thermogravimetric analysis (TGA), Fourier transform infrared spectroscopy (FTIR), and scanning electron microscopy (SEM) techniques. Our results revealed that the produced microstructures were able to endure sintering at 700 °C without collapsing, leading to scaffolds with 235 and 355 nm resolution and pore size, respectively. According to the TGA analysis, there was no significant mass loss beyond 600 °C. After sintering at 500 °C, the FTIR spectra showed the disappearance of the characteristic bands associated with the organic phase, and the presence of bands characteristic of the binary and ternary oxide systems and carbonate groups. The SEM images showed different morphologies of agglomerated nanoparticles with mean sizes of about 20 and 60 nm for ternary and binary systems, respectively. Our findings open the way towards precise control of bioglass scaffold fabrication with tremendous design flexibility.

## 1. Introduction

Glass additive manufacturing (AM) technologies are introducing new opportunities for the fabrication of photonic and optoelectronic devices. One of the most promising methods is two-photon polymerization (2PP) 3D printing. Indeed, it offers several advantages, such as the ability to produce glasses with high resolution up to 23 nm and good optical quality in flexible and complex geometries [1,2,3,4,5,6,7]. This technique involves the photopolymerization of a photoresist, where at least two photons are absorbed simultaneously. Photopolymerization reactions occur exclusively within the focal volume of a pulsed laser, due to the nonlinearity of this process [8]. Silica glass is among the most studied systems, thanks to its unique physico–chemical properties such as high chemical purity, thermal resistance, excellent optical transmission, and chemical durability [1]. The 2PP 3D print process for silica glasses involves the use of photocurable nanocomposite resins. At least, these consist of a liquid organic monomer containing a silica source and a photoinitiator. The most used silica sources are prefabricated nanoparticles [9,10] or molecular sol–gel precursors [4,11]. After 3D printing, a thermal treatment is required to convert the nanocomposite microstructures into silica glasses [4,9,10,12]. The recovered silica glasses are suitable for different applications such as micro-sensing [4] and optoelectronics [12]. However, various network-forming oxides or modifiers, such as P_2_O_5_ or CaO, can be incorporated inside the silica matrix to modify its properties [13,14]. These compositions offer opportunities for applications in various fields of biomedicine [15], including tissue engineering [16,17], dentistry [18,19], and bone regeneration in medicine [20,21,22]. These types of glasses have previously been fabricated in powder form using techniques such as sol–gel and melting processes [23,24,25,26]. However, it is difficult to form complex structure with bioglasses due to their stuffiness and brittleness [27]. AM presents great potential for scaffold fabrication with specific designs and tailored properties. Some bioglasses have already been made using different 3D printing techniques: fused deposition modeling (FDM), powder extrusion deposition (PED), direct ink writing (DIW), selective laser sintering (SLS), digital light processing (DLP), and stereolithography (SLA) [14,28,29,30,31,32]. Nevertheless, these 3D printing techniques have produced scaffold with a resolution of about 10 µm at best. However, improving bioglass AM resolution is still necessary to enhance the precision of scaffold manufacturing. A spatial resolution down to submicrometer scale is beneficial for precise control of scaffold pore structures [14]. Despite the great potential of 2PP 3D printing, to the best of our knowledge, such glasses have not yet been made using this technique. In the present study, we aim to demonstrate as proof of concept the possibility of 2PP 3D printing of scaffolds with submicron features, using two renowned oxide systems, namely SiO_2_-CaO and SiO_2_-CaO-P_2_O_5_. Here, we stress that the study of the bioactivity of such materials is out of the scope of this paper.

## 2. Materials and Methods

### 2.1. Syntheses of Organic Resin and Hybrid Photoresists

All the chemicals used were purchased from Sigma Aldrich (Sigma-Aldrich, Saint Quentin Fallavier, France) and were used as received. The sols of the SiO_2_-CaO and SiO_2_-CaO-P_2_O_5_ systems were synthesized by the sol–gel process using the following molecular precursors: tetraethyl orthosilicate (TEOS, ≥99%), calcium nitrate tetrahydrate (Ca(NO_3_)_3_·4H_2_O, ≥99%), and triethyl phosphate (TEP, >99%). We started by mixing TEOS, ethanol (EtOH, ≥99%), and deionized water in the following molar ratio: 1:2.4:1.45, under magnetic stirring for 30 min at room temperature (RT). Then, for the SiO_2_-CaO system, the calcium nitrate tetrahydrate was added and the mixture was stirred for 1 h to achieve a homogeneous solution. In the case of the SiO_2_-CaO-P_2_O_5_ system, the TEP was added secondly and the obtained solution was stirred for 30 min. Subsequently, the calcium nitrate tetrahydrate was introduced and the solution was stirred for 1 h. Finally, under stirring, 20 µL of nitric acid (HNO_3_ (2M), ≥99.99%) was added to each solution system to catalyze the sol–gel reactions. After 15 min, the recovered sols were clear, homogeneous, and transparent.

**Figure 1:**
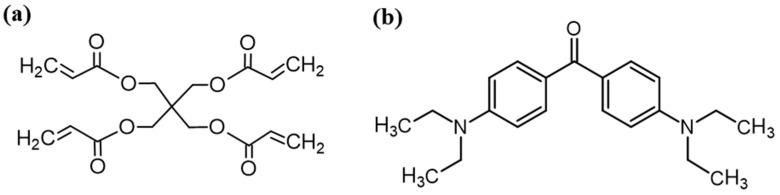
Chemical structures of pentaerythritol tetracrylate (**a**) and 4,4’-Bis(diethylamino) benzophenone (**b**) used for the preparation of organic resin.

The organic resin was synthesized by mixing the pentaerythritol tetracrylate (PETA) (98 wt%) and 4,4’-Bis(diethylamino) benzophenone (BDEB) (2 wt%) under magnetic stirring at room temperature. The PETA was used as a tetrafunctional acrylate monomer and the BDEB as an organic polymerization photoinitiator (Figure 1).

**Figure 2:**
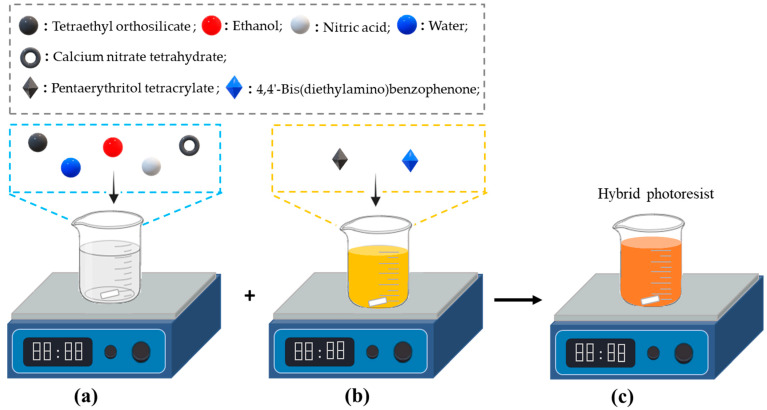
Schematic illustration of the synthesis procedure of the hybrid photoresist based on SiO_2_-CaO sol: (**a**) inorganic sol, (**b**) organic resin, and (**c**) hybrid photoresist.

The hybrid photoresist was prepared by solution mixing the SiO_2_-CaO sol (20 wt%) with the organic resin (80 wt%) under stirring for 15 min. A homogeneous, transparent, dark yellow solution was obtained (Figure 2). Following the same route, the hybrid photoresist with SiO_2_-CaO-P_2_O_5_ sol was also prepared.

### 2.2. Two-Photon Polymerization 3D Printing

**Figure 3:**
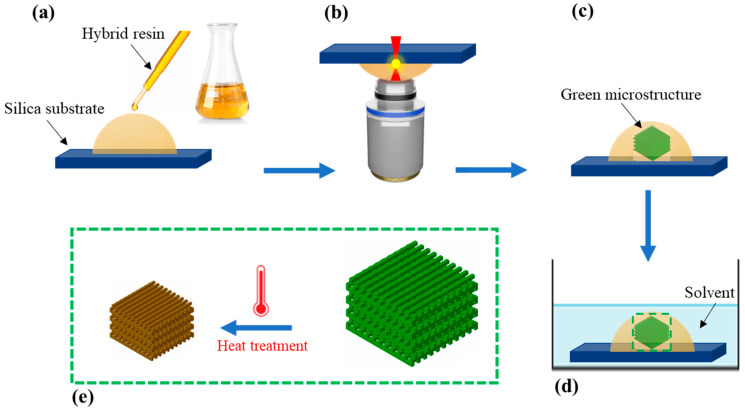
Different steps of the 3D printing process: (**a**) depositing a drop of hybrid photoresist, (**b**) 3D printing using dip-in laser lithography configuration, (**c**) end of 3D printing, (**d**) development, (**e**) heat treatment of the printed microstructure.

The 3D printing of the microstructures was performed using the 2PP technique on fused silica substrates (3D SF DiLL, 25 mm × 25 mm × 0.7 mm, from Nanoscribe GmbH, Eggenstein-Leopoldshafen, Germany). After designing the 3D model, the file (.STL) was imported into Describe software (version 2.7, Nanoscribe GmbH, Karlsruhe, Germany). The 3D printing parameters were adjusted in Describe to generate the job file (.GWL). Figure 3 shows the different steps of our 3D printing process. Firstly, a drop of the hybrid photoresist was placed on the fused silica substrate (Figure 3a). Then, the microstructures were printed using a Photonic Professional GT+ commercial printer (Nanoscribe GmbH, Karlsruhe, Germany) in the dip-in laser lithography (DiLL) configuration (Figure 3b). When the 3D printing was complete (Figure 3c), a development step was required to remove the unpolymerized photoresist. This consisted of immersing the substrate in propylene glycol monomethyl ether acetate (PGMEA) for 10 min followed by isopropyl alcohol (IPA) for 5 min, to recover the green microstructures (Figure 3d). Finally, the 3D-printed microstructures were heat treated under air at 700 °C, as detailed hereinafter. The 3D printer employed a femtosecond fiber laser source with a wavelength centered at 780 nm, a mean power of about 50 mW, a pulse duration of 100 fs, and a repetition rate of 80 MHz. A 63x/NA1.4 objective lens was used (from Zeiss society, Oberkochen, Germany).

### 2.3. Heat Treatment Process

**Figure 4:**
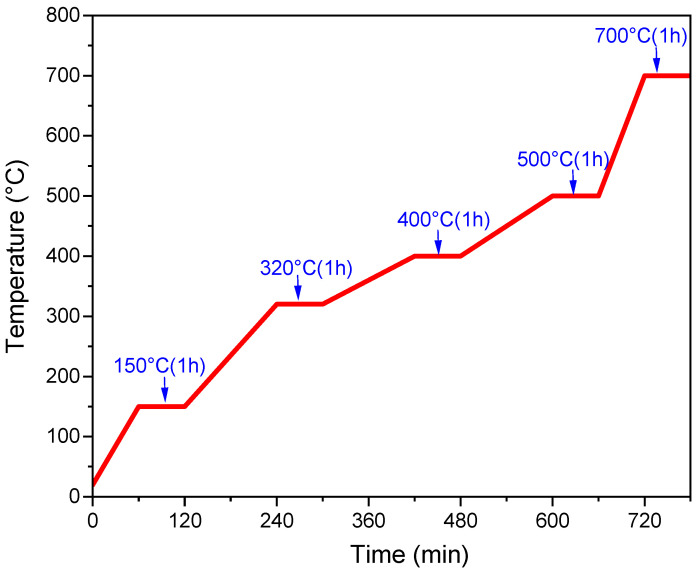
The multi-step heat treatment protocol.

The heat treatment was performed under air from RT to 700 °C in an LHTCT 01/16 furnace with lift Door (Nabertherm GmbH, Lilienthal, Germany). The samples were heat treated according to the stepwise heating protocol detailed in Figure 4. Different heating rates were used: 150 °C (2.2 °C/min), 320 °C (1.4 °C/min), 400 °C (0.7 °C/min), 500 °C (0.8 °C/min), and 700 °C (3.3 °C/min). After that, the substrates were removed from the furnace and left to cool down to room temperature.

### 2.4. Structural and Morphological Characterizations

For thermogravimetric analysis (TGA), a drop of the hybrid photoresist was deposited on a silica glass substrate and exposed to UV radiation. After UV curing using a Form Cure (Formlabs, Somerville, MA, USA), the powdered sample was recovered from the substrate. The TGA was carried out under air atmosphere using a Netzsch STA 2500 instrument (Netzsch, Selb, Germany), from RT to 1000 °C at a heating rate of 5 °C/min. The FTIR spectra of the UV-cured hybrid photoresists before and after heat treatment at 500 and 700 °C were recorded using a Nicolet 300 spectrometer (Thermo Fisher Scientific Inc., Waltham, MA, USA) equipped with a single reflection diamond ATR accessory. The scanning electron microscopy (SEM) images of the 3D-printed microstructures were obtained using two Field Emission Scanning Electron Microscopes (FE-SEM): SU8230 and SU5000 (Hitachi High Technologies, Tokyo, Japan). The optical images were obtained using Eclipse LV100 microscope (Nikon Corporation, Tokyo, Japan).

## 3. Results and Discussion

### 3.1. 3D Printed Microstructures

**Figure 5:**
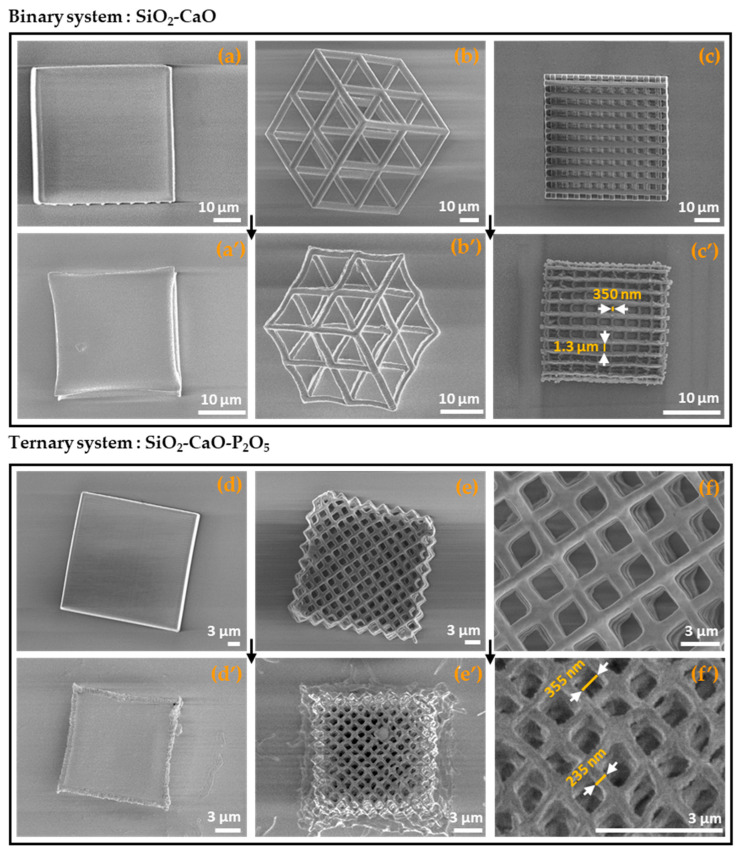
3D-printed microstructures of binary (**a**–**c**) and ternary (**d**–**f**) systems before (**a**–**f**) and after heat treatment at 700 °C (**a′**–**f′**), respectively; (**f**) zoomed image of (**e**) and (**f′**) zoomed image of (**e′**).

Figure 5 shows the 3D printed microstructures using both binary (a–c) and ternary (d–f) hybrid photoresists, before and after heat treatment at 700 °C. All the microstructures were 3D printed according to the following printing parameters: laser power (100%), scan speed (10,000 μm/s), and slicing/hatching distances (0.3 and 0.2 μm, respectively). The choice of sintering temperature was based on the results of the TGA analysis (see the Section 3.3). Although the thermograms showed a stabilization of mass loss at 600 °C due to the degradation of the organic phase, the microstructures were heat treated up to 700 °C to test their thermal resistance.

We also successfully 3D printed microcubes (Figure 5a,d) and more complex microstructures such as superlattice (Figure 5b), woodpile (Figure 5c), and cube–vertex–centroid (Figure 5e). After sintering at 700 °C, we observed that all the microstructures retained their shapes (Figure 5a′–f′) with slight deformation. Furthermore, we stress that binary microstructures showed higher thermal resistance to sintering at 700 °C compared with those of the ternary system. That was why we heat treated the microstructures for 1 h and 3 h for the ternary and binary systems, respectively. This result could be related to the presence of phosphorus in the composition of the ternary system. Furthermore, 3D objects with submicrometer features were produced, as shown in Figure 5c′,f′. According to this Figure, the resolutions reached for the binary and ternary systems were 350 nm and 235 nm, respectively. We note that such resolutions could be improved through further optimization of the resin composition and 3D printing parameters. Moreover, a scaffold pore size of about 355 nm was also obtained (Figure 5f′), which has never been achieved using other 3D-printing techniques, to the best of our knowledge. These results show that our approach can be used to develop adapted 3D scaffold structures for biomedical applications where scaffolds with small pore sizes are needed [33,34,35,36,37].

In addition, using optical microscopy to measure the size of printed microcubes before and after sintering at 700 °C, we observed that both the binary and ternary systems presented a close shrinkage rate of about 55–57% (Table 1). We note here that both the SiO_2_-CaO and SiO_2_-CaO-P_2_O_5_-based photoresists contained similar amounts of the organic resin. The obtained results show that the shrinkage was determined by the amount of the organic resin in the hybrid photoresist.

### 3.2. Morphological and Structural Analyses

#### 3.2.1. Morphological Analysis

**Figure 6:**
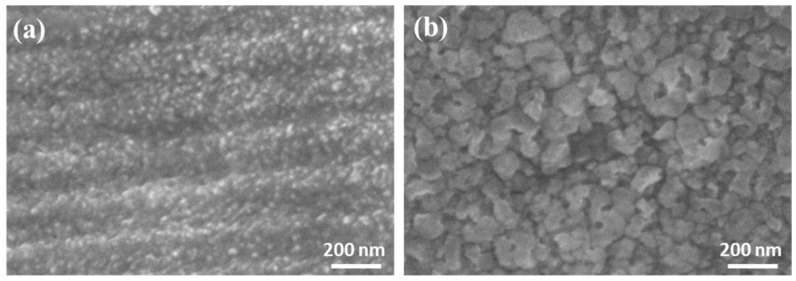
Surface morphology SEM images of SiO_2_-CaO-P_2_O_5_ (**a**) and SiO_2_-CaO (**b**) oxide system microcubes after heat treatment at 700 °C.

SEM micrographs were taken from the surfaces of 3D-printed microcubes of the binary and ternary systems after sintering at 700 °C. The images are shown in Figure 6. They reveal different morphologies depending on the composition of the material. Indeed, in the case of the ternary system (Figure 6a), the SEM image showed that it was made of an agglomeration of finer particles with an average particle size of about 20 nm. However, regarding the binary system (Figure 6b), the SEM micrograph revealed the presence of agglomerated particles with irregular shapes and a rough texture with an average particle size of about 60 nm. The presence of smaller nanoparticles (20 nm) in the case of the ternary system compared with the binary (60 nm), could be attributed to the presence of phosphorus in the composition of the ternary system.

#### 3.2.2. FTIR Analysis

**Figure 7:**
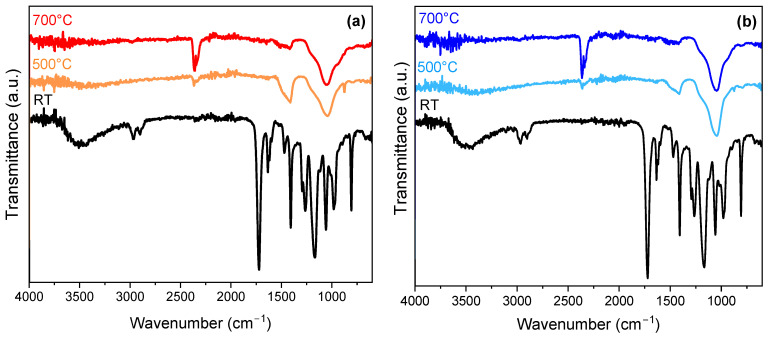
FTIR spectra of UV-treated hybrid photoresists before and after heat treatment at 500 and 700 °C for 1 h: (**a**) SiO_2_-CaO and (**b**) SiO_2_-CaO-P_2_O_5_.

The FTIR spectra of the hybrid photoresists before and after heat treatment at 500 and 700 °C are shown in Figure 7. Absorption bands associated with the organic phase in the UV-treated hybrid photoresists were observed, as summarized in Table 2. Moreover, the wide band peaking at 3500 cm^−1^ originated from the -O-H stretching vibrations of hydroxyl groups and the stretching vibrations of free silanol groups [38]. After heat treatment at 500 °C, we observed the disappearance of absorption bands associated with the organic phase, and the FTIR spectra showed bands characteristic of the SiO_2_-CaO binary or SiO_2_-CaO-P_2_O_5_ ternary systems (Figure 7a,b). The typical bands of silica-based systems at around 798 and 1046 cm^−1^ correspond, respectively, to the symmetric and asymmetric stretching modes of Si–O–Si [4,39]. The observed vibrational band at 940 cm^−1^ was assigned to the stretching vibrations of the nonbridging Si-O (Si-O-NBO) and associated with the presence of calcium ions [38]. With regard to the ternary system, we do not exclude the presence of a band associated with the P–O bond around 1045–1090 cm^−1^, although this was masked by the broad silicate band [40]. We noted the appearance of two absorption bands peaking around 1420 and 1520 cm^−1^, attributed to the anti-symmetric stretching of C-O in carbonate groups CO_3_^2-^ [38,39,40]. The presence of these groups was associated with calcium and its reaction with the residual carbone [38] or the atmospheric CO_2_ [40]. The incorporation of carbonate groups provides the advantage of improving the solubility and resorbability processes that occur in bone [41,42]. The double peak around 2340 and 2363 cm^−1^ can be attributed to adsorbed CO_2_, as observed in similar systems [39,43,44]. After the heat treatment at 700 °C, compared with 500 °C, we noted that the band attributed to carbonate groups decreased while that associated with the absorbed CO_2_ increased. Morever, the band at 3500 cm^−1^ completely disappeared after heat treatment at 700 °C, which was due to the elimination of hydroxyl groups.

### 3.3. TGA

**Figure 8:**
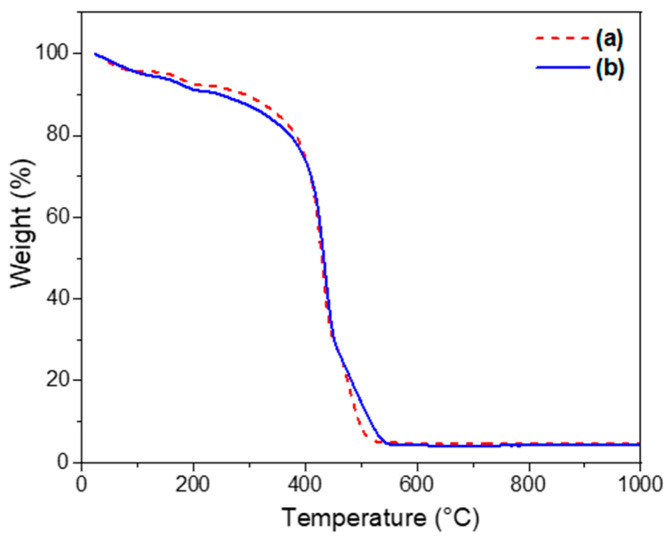
TGA curves of the UV-cured hybrid photoresists comprising (a) binary SiO_2_-CaO and (b) ternary SiO_2_-CaO-P_2_O_5_ systems.

Figure 8 shows the TGA curves of the UV-cured hybrid photoresists; note that both SiO_2_-CaO and SiO_2_-CaO-P_2_O_5_ systems present similar TGA profiles. Three stages of mass loss were observed in the TGA curves. The first mass loss of about 8% ranged from 25 to 200 °C and was attributed to the removal of residual water and ethanol [38,47,48]. In the second range, 200–550 °C, the weight loss was about 87.4%, which was assigned to thermo–oxidative degradation of highly cross-linked acrylic polymers and the thermal decomposition of residual nitrates [4,47]. The weight loss shown in thermogram (b) was slightly delayed compared with that in (a), which could have been related to the presence of phosphorus. In the range 550–700 °C, the third mass loss was about 0.4%, which could have been associated with the thermal degradation of residual organics and condensation reactions. For this reason, we stopped the thermal treatment of our samples at 700 °C.

## 4. Conclusions

In summary, new hybrid photoresists suitable for 2PP additive manufacturing of multicomponent oxide systems were successfully synthesized via a combination of organic resin and sol–gel sols. It was found that the 3D-printed microstructures endure a sintering temperature up to 700 °C leading to 3D scaffolds with submicrometer features and a resolution of about 235 nm with pore size of about 355 nm. After sintering at 700 °C, it was shown that the obtained binary (SiO_2_-CaO) and ternary (SiO_2_-CaO-P_2_O_5_) oxide systems, incorporating carbonate groups, present different morphology. They were formed of agglomerated nanoparticles with size of about 60 and 20 nm, for the binary and ternary system, respectively. Our findings open new opportunities for the precise fabrication of complex multicomponent oxide structures such as multiscale interconnected porous scaffolds for bone regeneration in the biomedical field.

## Figures and Tables

**Table 1:** 3D-printed microcube dimensions for binary and ternary systems before and after heat treatment at 700 °C, and their shrinkage rates.

System	Side Length (RT)	Side Length (700 °C)	Shrinkage Rate (%)
Binary system	30.2 µm	13.0 µm	57
Ternary system	30.5 µm	13.7 µm	55

**Table 2:** FTIR bands assigned to the organic phase in the UV-treated hybrid photoresists [4,45,46].

Binary System (cm^−1^)	Ternary System (cm^−1^)	Attribution
2964	2964	ν (C-H)
2900	2902	ν (C-H)
1722	1722	ν (C=O)
1634	1634	ν(C=C)
1635	1635	ν (C=C)
1619	1918	ν (C=C)
1472	1470	δ (C-H)
1408	1407	δ (C-H)
1296	1294	γ_twisting_ (CH_2_)
1263	1263	ν (C-O-CH_3_)_ester_
1169	1169	ν (C-C)
1059	1059	ν (C-O)
982	981	ν (Si-OH)
806	806	δ (=C-H)

## Data Availability

The data presented in this study are available on request from the corresponding author.
